# Supplementary material for: CXCR6+ CD127− Tr1 Cells Balance Immunity and Persistence in Plasmodium falciparum Infection
Source: bioRxiv. 2025 Sep 18:2025.09.16.676649. Preprint. [Version 1] doi: 10.1101/2025.09.16.676649 (PMC12458128; doi:10.1101/2025.09.16.676649)
Supplement: Supplement 1 [file media-1.pdf]

Supplementary Figures

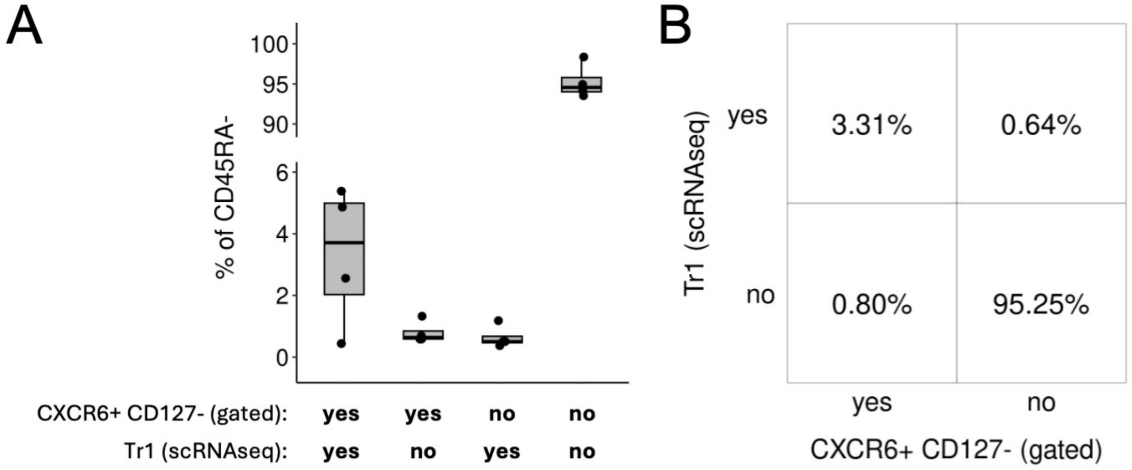

Fig. S1. Confusion matrix (and underlying data) describing the effectiveness of CXCR6+ CD127- as a gating strategy for identifying Tr1 cells. (**A**) Box plots displaying the percent of CD45RA- (memory) CD4+ T cells that are Tr1 or non-Tr1 and that fall inside or outside of the CXCR6+ CD127- gate. A break in the y-axis is used to improve data visualization. (**B**) Confusion matrix reporting averages of the percentages in 'A'.

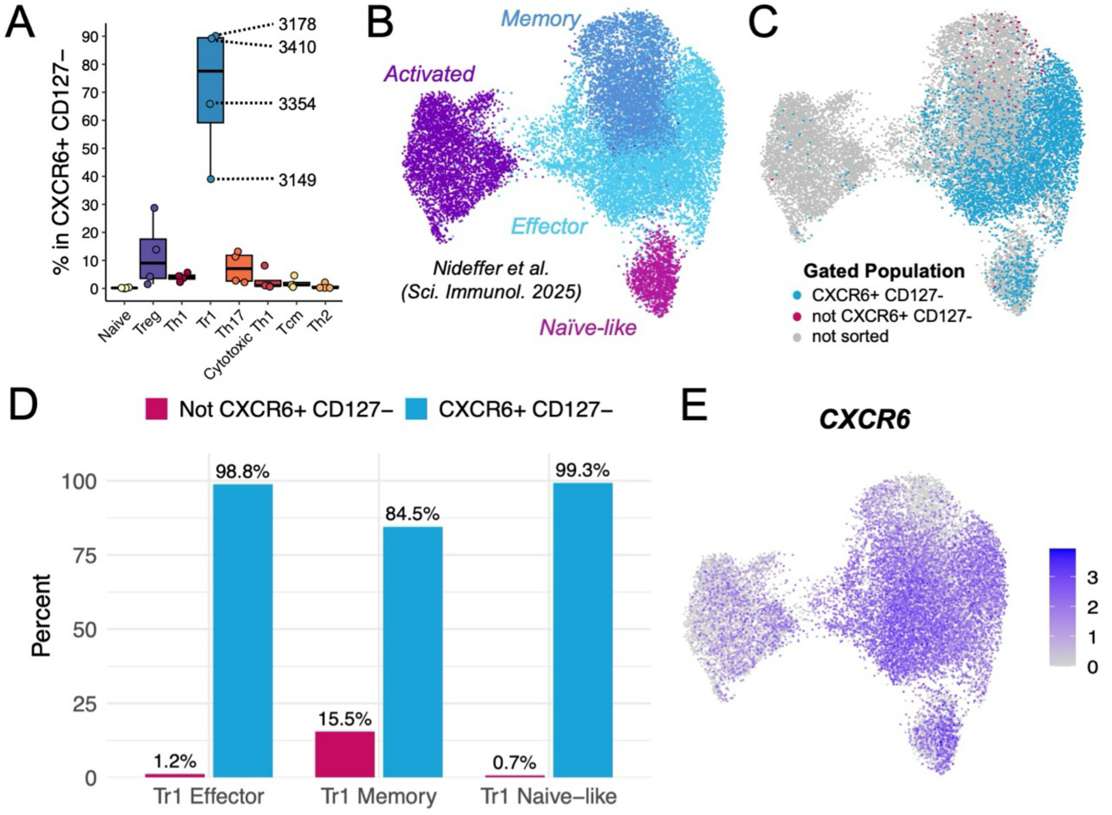

Fig. S2. Quantification of the heterogeneity amongst CXCR6+ CD127- memory CD4+ T cells. (**A**) The percentage of CXCR6+ CD127- memory CD4+ T cells that belong to different subsets (defined by scRNAseq). Numbers indicate which data points correspond to each of the four donors. (**B**) UMAP from our prior study depicting the heterogeneity amongst Tr1 cells. (**C**) The same UMAP as in 'B' colored according to whether or not cells were sorted as CXCR6+ CD127-. (**D**) Bar graph quantifying the percentage of a given Tr1 cell subset that was captured (or not captured) by the CXCR6+ CD127- gate. (**E**) The same UMAP as in 'B' colored according to expression of CXCR6.

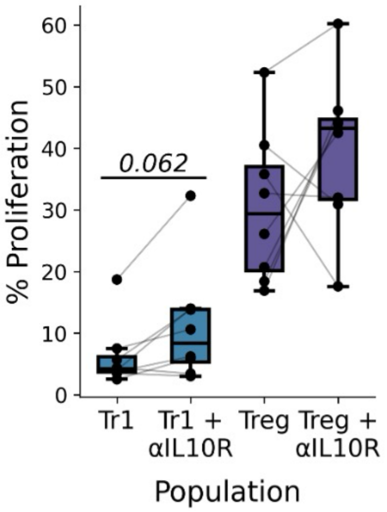

Fig. S3. Tr1 and conventional Treg proliferation in the presence of IL-10 receptor blockade. Cells were incubated for 4 days in the presence of αCD3 and αCD28 as part of a suppression assay. Lines connect different conditions using cells that that were derived from the same donor. Paired T tests were performed comparing conditions with and without IL-10 receptor blockade.

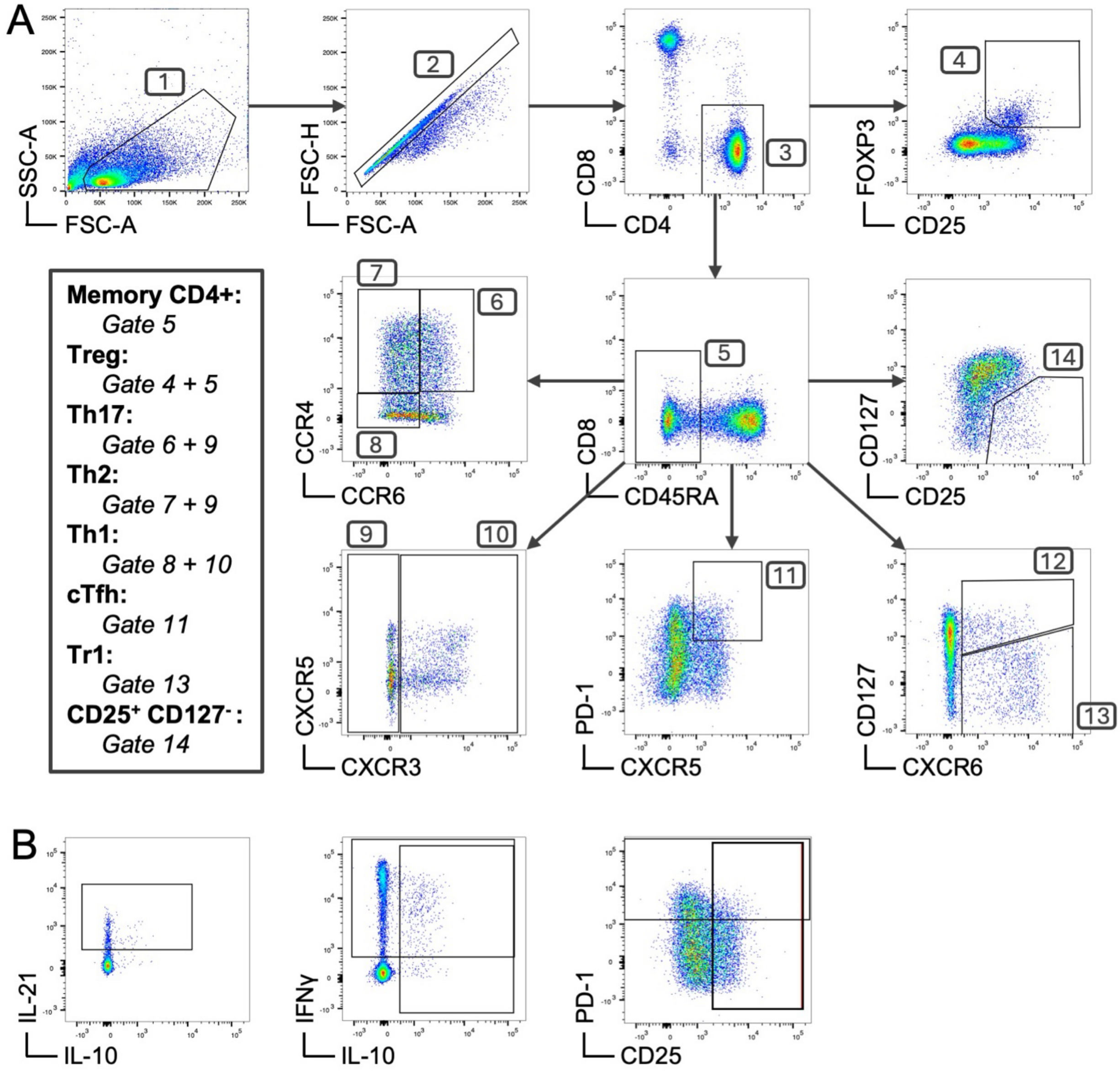

Fig. S4. Gating of CD4+ T cell subsets and effector molecules. (**A**) Flow cytometry plots depicting the gating strategy for identifying different cellular populations. (**B**) Flow cytometry plots showing staining of key cytokines and surface-expressed effector molecules.

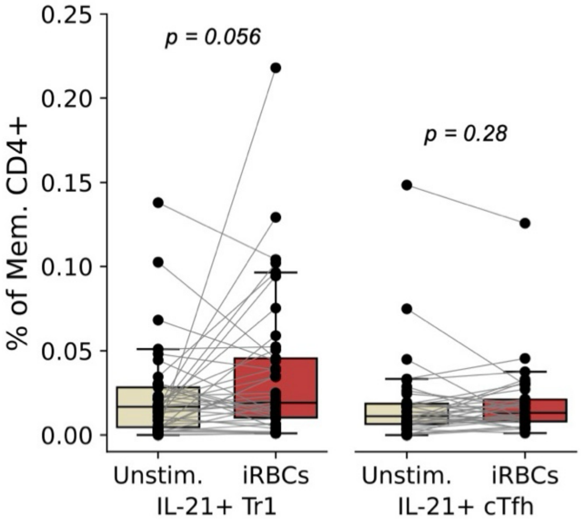

Fig. S5. IL-21 expression by Tr1 and cTfh cells following stimulation with iRBCs. The percentage of memory CD4+ T cells that were CXCR6+ CD127- Tr1 (left) or CXCR5+ PD-1+ cTfh and expressed IL-21 in response to iRBC stimulation (compared to unstimulated). Significance was determined via paired T tests.

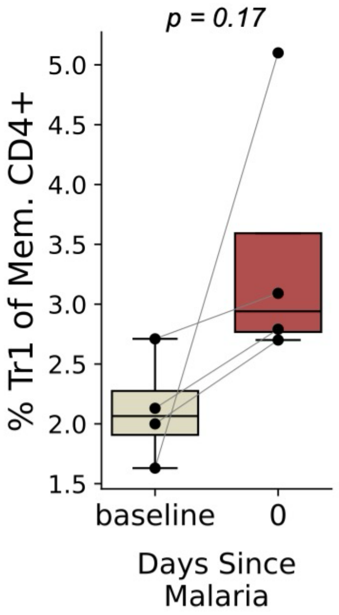

Fig. S6. Expansion of Tr1 cell frequencies in Ugandan adults diagnosed with malaria. Tr1 frequencies were measured as a proportion of memory CD4+ T cells at a baseline timepoint prior to infection and at the time of diagnosis. Significance was determined via a paired T test.

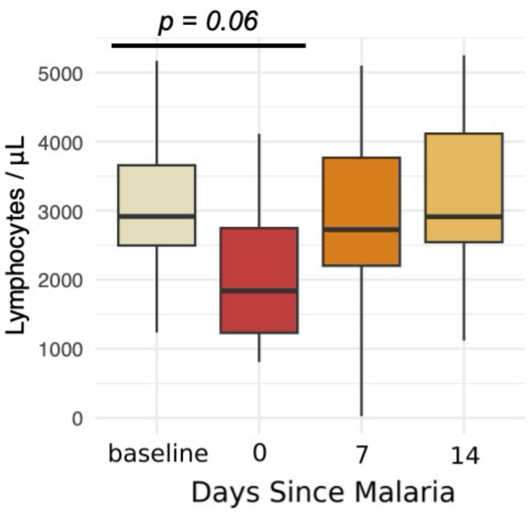

Fig. S7. Absolute lymphocyte counts in the context of malaria. Lymphocyte counts were measured before, during, and after symptomatic malaria. Significance was determined using a paired T test comparing mean counts at baseline compared to day 0.

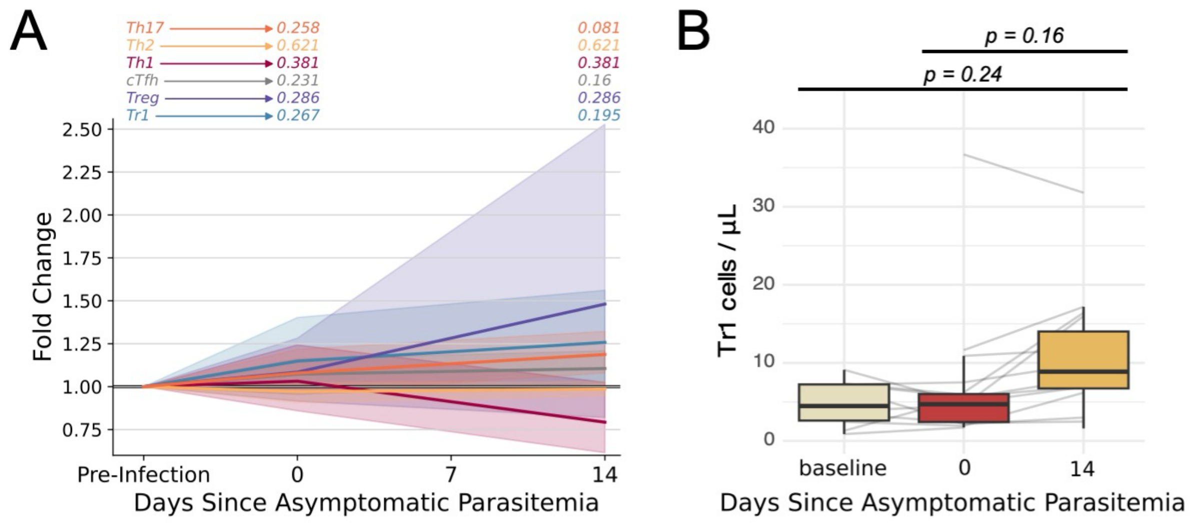

Fig. S8. Infection dynamics of memory CD4+ T cells populations during asymptomatic parasitemia. (**A**) Fold change (compared to the pre-infection baseline) in cell frequencies following the detection of asymptomatic parasitemia as determined by flow cytometry. P-values are displayed above each sample timepoint for each population and represent pair-wise comparisons to baseline. (**B**) The absolute counts of Tr1 cells in peripheral blood before, during, and after asymptomatic parasitemia.

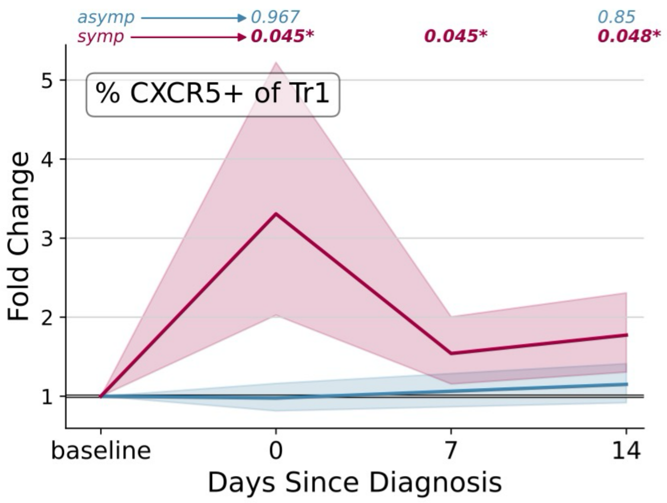

Fig. S9. Fold change in the percentage of Tr1 cells expressing CXCR5 following symptomatic malaria or asymptomatic parasitemia. p-values are displayed above each sample timepoint for each infection type and represent pair-wise comparisons to baseline.

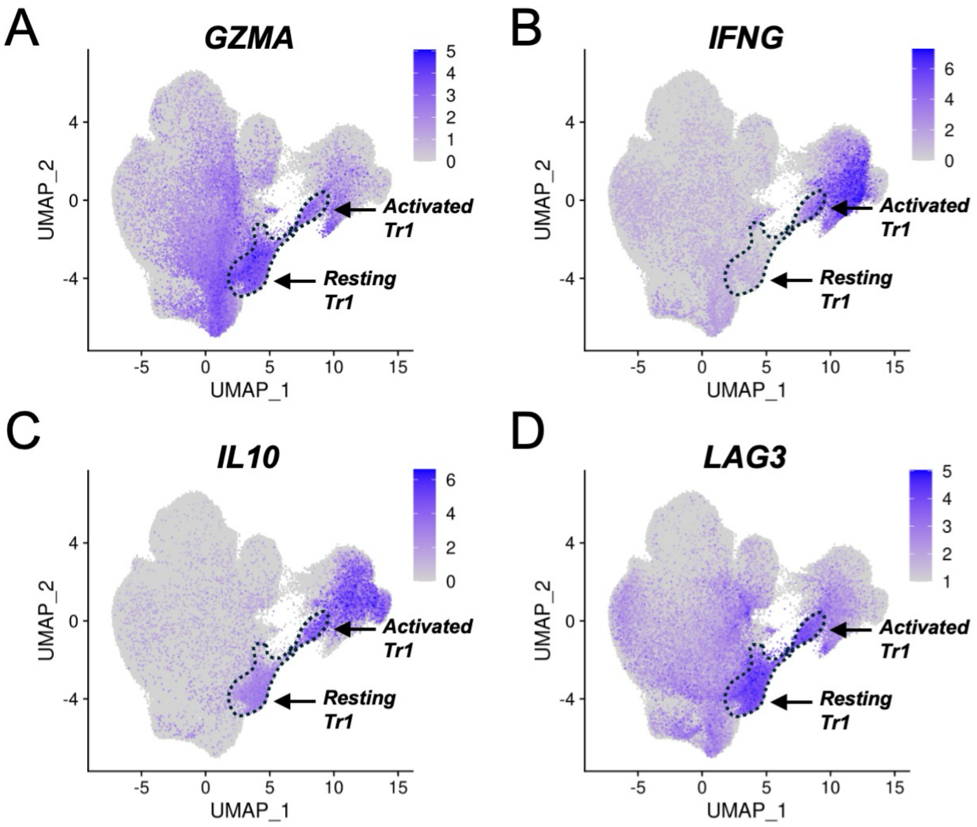

Fig. S10. Expression of Tr1-associated genes by memory CD4+ T cells. (**A-D**) UMAPs with the same architecture as Fig. 1A colored according to the expression levels of *GZMA* ('A'), *IFNG* ('B'), *IL10* ('C'), and *LAG3* ('D'). The dashed lines highlight resting and activated Tr1 populations.

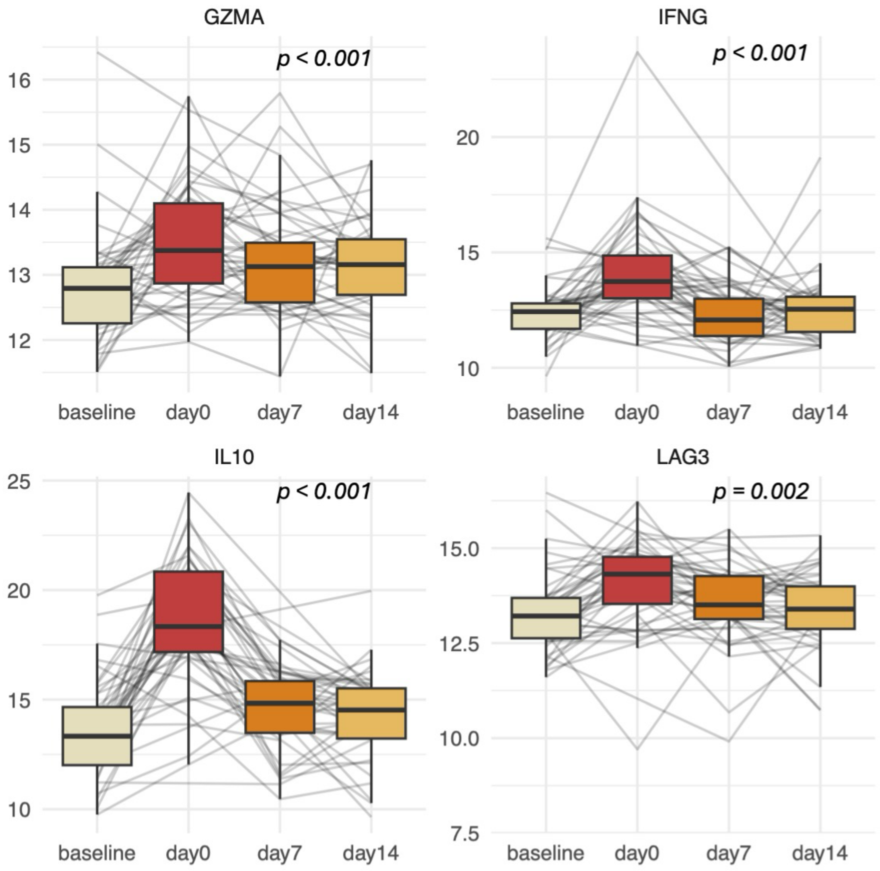

Fig. S11. Tr1 plasma protein abundance in the context of symptomatic malaria. The abundance of granzyme-A, IFNγ, IL-10, and LAG-3 in plasma samples (determined by NULISA) collected before, during, and after symptomatic malaria or asymptomatic parasitemia. All p-values shown were adjusted to correct for multiple hypothesis testing. Unless the p-value is explicitly reported, p-value < 0.05 = *; p-value < 0.01 = **; p-value < 0.001 = ***.

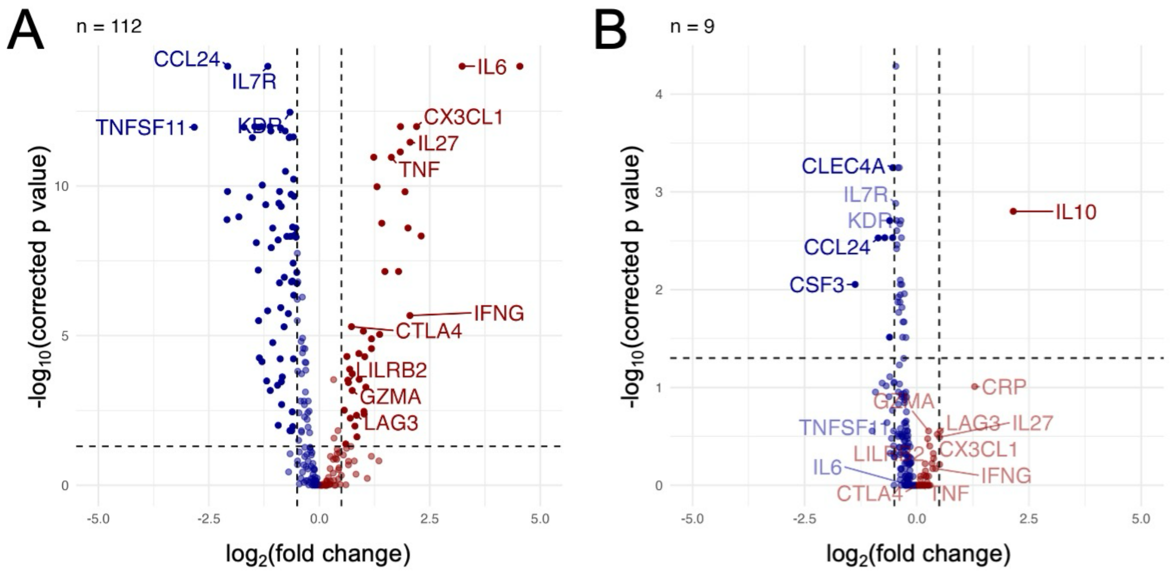

Fig. S12. Differential abundance of plasma proteins measured by NULISA during symptomatic and asymptomatic infections. (**A-B**) Volcano plots depicting proteins that are differentially abundant at diagnosis with symptomatic malaria ('A') or asymptomatic parasitemia ('B') versus a pre-infection baseline.

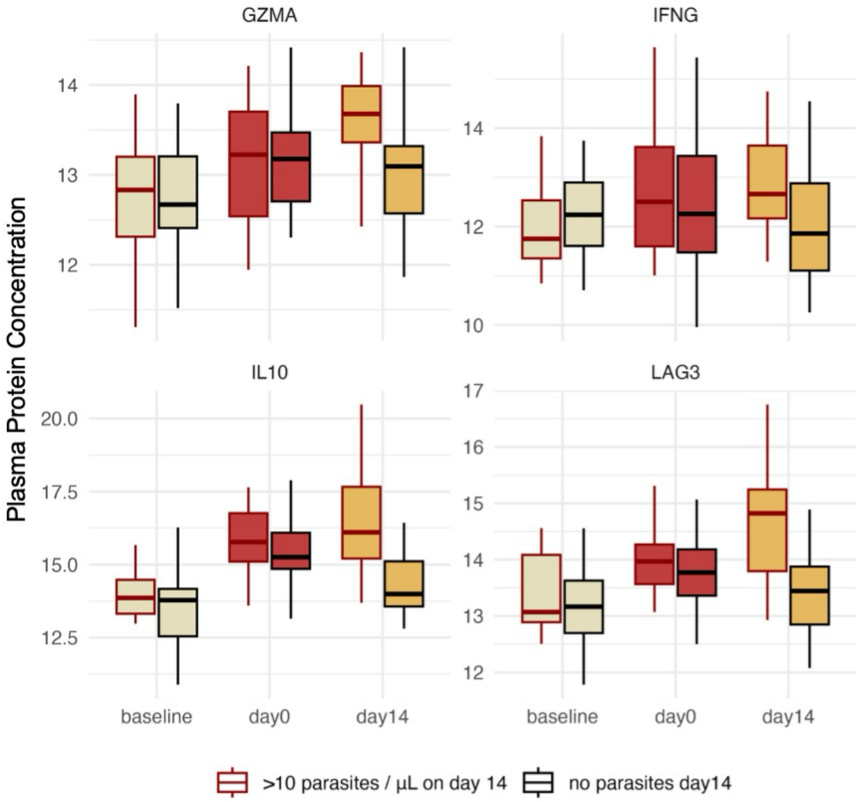

Fig. S13. The effect of persistent parasitemia on plasma concentrations of Tr1 effector molecules. The abundance of granzyme-A, IFNγ, IL-10, and LAG-3 in plasma samples (determined by NULISA) collected before during and after asymptomatic parasitemia, stratified by whether the subject tested parasitemia positive or negative at day 14.

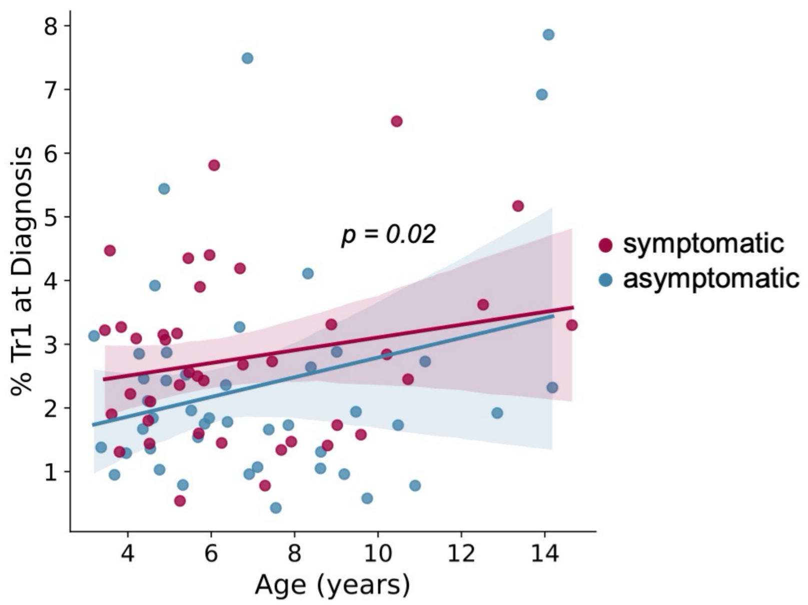

Fig. S14. Correlation between age and Tr1 frequency at the time of diagnosis. Separate linear regressions are displayed for symptomatic and asymptomatic infections. Shaded regions represent 95% confidence ranges. The displayed p-value represents the significance of an effect of age on Tr1 frequency in a generalized linear model that includes infection type (symptomatic or asymptomatic) as an independent categorical variable.

Table S1. Characteristics of MUSICAL study participants.

**Characteristic**	
**Age in years at enrollment**	4.3 (1.8-13.5)
**Female sex, n (%)**	20 (41.7%)
**Mean parasite density at time of symptomatic infection, SD**	18929.2 (19955.39)
**Mean parasite density at time of asymptomatic infection, SD**	3453.3 (6038.5)
**Sickle cell status***	
**HbAA, n/N(%)**	36/46 (75.0%)
**HbAS, n/N(%)**	10/46 (20.8%)
**Incidence of malaria per person year over period of cohort (2020-2023)**	1.96

*2 individuals with missing Hb genotype data

Table S2. Univariate and multivariate analyses of the relationship between Tr1 frequencies and the future incidence of malaria or the duration of incident asymptomatic infections.

	Future incidence of symptomatic malaria over 2 years				Duration of incident asymptomatic infection in days[1]			
	Univariate		Multivariate[2]		Univariate		Multivariate[2]	
	IRR (95% CI)	P	IRR (95% CI)	P	Coef (95% CI)	P	Coef (95% CI)	P
**Age in years**	0.89 (0.80-0.98)	0.02	0.87 (0.78-0.97)	0.01	0.38 (-3.4-4.2)	0.84	2.2 (-2.5-6.9)	0.36
**Log10 parasite density at diagnosis**	1.06 (0.82-1.37)	0.67	1.02 (0.78-1.32)	0.89	4.15 (-6.9-15.2)	0.46	7.36 (-1.9 – 16.6)	0.12
**%CXCR6+CD127- Tr1 prior to infection**								
**Group 1, n=22 (0.43%-1.5%)**	Ref	Ref	Ref	Ref	Ref	Ref	Ref	Ref
**Group 2, n=40 (1.5%-3.08%)**	1.43 (0.83-2.48)	0.20	1.39 (0.76-2.55)	0.29	23.7 (-3.2-50.6)	0.085	27.3 (-3.5-58.2)	0.08
**Group 3, n=23 (3.1% - 7.86%)**	0.77 (0.37-1.59)	0.48	0.76 (0.37-1.59)	0.47	33.0 (-6.9-3.0)	0.10	42.5 (3.6-81.4)	0.032
**%CXCR6+CD127- Tr1 at time of diagnosis**								
**Group 1, n=22 (0.43%-1.5%)**	Ref	Ref	Ref	Ref	Ref	Ref	Ref	Ref
**Group 2, n=40 (1.5%-3.08%)**	0.74 (0.33-1.62)	0.45	0.74 (0.35-1.57)	0.44	15.5 (-14-45.1)	0.31	11.23 (-21.5-44.0)	0.50
**Group 3, n=23 (3.1% - 7.86%)**	0.38 (0.18-0.80)	0.011	0.38 (0.18-0.80)	0.011	51.8 (17.4-86.3)	0.003	52.67 (18.9-86.5)	0.002

IQR:
[1]Incidence infections defined by AMA1 amplicon sequencing.
[2]Multivariate models adjusting for age in years and log_{10} parasite densities (detected by quantitative PCR)
